# Supplementary material for: Tuning of Liver Sieve: The Interplay between Actin and Myosin Regulatory Light Chain Regulates Fenestration Size and Number in Murine Liver Sinusoidal Endothelial Cells
Source: Int J Mol Sci. 2022 Aug 30;23(17):9850. doi: 10.3390/ijms23179850 (PMC9456121; doi:10.3390/ijms23179850)
Supplement: Supplementary file 1 [file ijms-23-09850-s001.zip › ijms-1819581-supplementary.pdf]

SUPPLEMENTARY INFORMATION

The role of myosin light chain in the regulation of fenestration size and number in murine liver sinusoidal endothelial cells

Bartlomiej Zapotoczny[1,2], Karolina Szafranska[1], Malgorzata Lekka[2], Balpreet Singh Ahluwalia[3], Peter McCourt[1]

Contents

1. Endocytosis and viability 1
2. Structured illumination microscopy (SIM) in the determination of level of pMLC and ppMLC in LSEC treated with selected agents 3
3. Semi-quantitative LSEC morphology analysis using overview mode in SEM 8
4. Relaxed versus stretched fenestrations 12
5. The effect of Calyculin A on the morphology of LSEC 13
6. The list of reagents used in the experiments. 15

1. Endocytosis and viability

MLC is a crucial point in the pathways regulating myosin contraction and actin (de)polymerisation. The inhibitors used here can be toxic and their effect on fenestrations might related to harmful side effects, especially in higher concentrations. Therefore, we performed various functional tests to assess the condition of cells in addition to examining their morphology.

1. Formaldehyde-treated serum albumin (FSA) particles in the process of LSEC endocytosis

It was demonstrated that healthy LSEC scavenge formaldehyde-treated serum albumin (FSA) particles in the process of endocytosis [1–3]. Both degradation and/or uptake of FSA decrease both in ageing[4,5] and as after treatment with hepatic toxicants[5]. Therefore, the FSA endocytosis can be used as an indicator of LSEC functional viability. FSA is taken up via stabilin 1 and 2 receptors and later degraded the endo-lysosomal compartments[6–8]. The degradation rate is highly susceptible to any distortion and the reduction is a manifestation of toxic side effects of the tested compounds. The reduction of degradation without reduction of uptake may also suggest an intracellular transportation issues as FSA is actively transported along the microtubule toward the perinuclear regions. Simone-Santamaria et al. also showed that endocytic capacity is independent from the fenestration parameters[4].

Here, we showed that the degradation of FSA decreased by 50% after treatments with CytB, 10 µM ML-7, and 10 nM CalA challenge (**Figure S1, A**). Degradation of endocytosed FSA was reduced by nearly

80% with 20 µM ML-7 and reduced to zero with 100 nM CalA. KN93, Y27632, blebbistatin and 1 nM CalA did not affect endocytosis. The cumulative effect of an inhibitor with CytB resulted in a cumulative reduction of degraded FSA; additional reduction of degraded FSA was observed for 10 nM and 100 nM CalA.

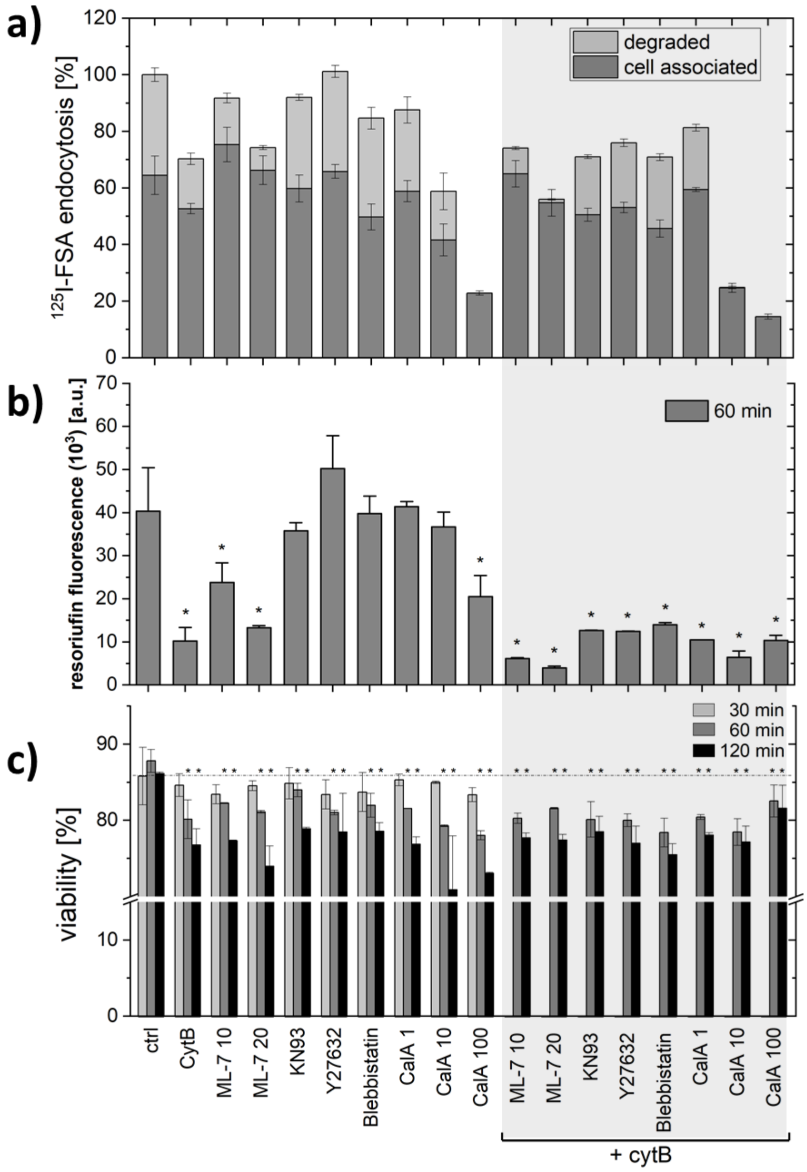

Figure S1 | Endocytosis activity (a), mitochondrial activity (b), and viability (c) of LSEC after inhibitor treatment. a) The chart shows the cell associated and degraded ^{125}I-FSA from LSEC treated for 1 h with selected inhibitors. b) The chart presents the amount of resazurin reduced to resorufin, corresponding to the mitochondrial metabolic activity of LSEC. * p<0.01. c) The effect of selected inhibitors on viability of LSEC, presented as LDH release from discontinuous cell membranes. The same set of inhibitors was used in all experiments.

2. Resazurin/resorufin assay

The assay is based on the reduction of resazurin (oxidized, non-fluorescent form) to resorufin (reduced, fluorescent form, Ex/Em of 530-560 / 590 nm) by the mitochondrial respiratory chain. The rate of that

conversion can be used as an indicator of the mitochondrial function and viability. LSEC ATP metabolism is flexible and can be flexibly switched between oxygen dependent mitochondrial respiration and oxygen independent glycolysis [9]. It is most probably related to the low oxygen conditions within the liver sinusoids [10]. Cytochalasin promotes depolymerization and rearrangement of actin cytoskeleton, which is ATP dependent, but at the same time decrease mitochondrial function. This confirm that LSEC can efficiently produce ATP independent of mitochondria. Therefore, resazurin assay can be used as an indicator of mitochondrial function of LSEC rather than cell viability.

We used the resazurin assay to test the mitochondrial metabolic activity of LSEC treated with inhibitors for 60 min. Resazurin (non-fluorescent) is reduced to resorufin (fluorescent) in the respiratory chain that allows assessment of mitochondrial activity. We observed a significant reduction after CytB, ML-7, and 100 nM CalA treatment (**Figure S1, B**). Moreover, all combinations with CytB resulted in severe reduction of the observed fluorescence signal. Y27632 produced an insignificant increase in mitochondrial activity.

3. Lactate dehydrogenase (LDH) test

Finally, we assessed LDH release from cells treated with inhibitors. LDH is released from damaged cells and is frequently used as a cell viability parameter. A small fraction of LSEC were damaged during the isolation procedure or died during the first hour after seeding. Applying the LDH assay, we observed 85% viability of control cells (normalized to cells lysed using 5% Triton X-100) (**Figure S1, C**). After the treatment with the inhibitors, we observed a gradual increase of the LDH release (reduced viability) for all inhibitors, down to 75% viability after 2h of 20 µM ML-7 and 100 nM CalA treatment. The result is expected, as the phosphorylation of MLC is crucial for cell fate, including cell proliferation, differentiation, and apoptosis [11,12]. After 60 minutes of treatment the viability loss was less than 5% for all inhibitors except for 10 nM and 100 nM CalA. Therefore, we used 60 min treatment of all inhibitors, except for CalA, where 30 min treatment was used to analyse LSEC morphology. The mean fenestration lifespan was previously shown to be <20 minutes [13]. Therefore, the effects of the inhibitors should be manifest after 30-60 minutes, if they are directly involved in the fenestration formation.

2. Structured illumination microscopy (SIM) in the determination of level of pMLC and ppMLC in LSEC treated with selected agents

Investigated LSEC are primary cells. Cell viability is affected due to long cell isolation and about 10% of cells dies during the seeding or during the first hours of culture (**Figure S1**). As presented in Figure S6, not all damaged cells can be rinsed from the culture and some of them remain in culture during the analysis. The process of apoptosis activates phosphorylation of MLC and investigating the whole sample with Western Blot or plate reader would uniform the signal and hamper detailed investigation of differences. Here, we apply SIM to:

a) find representative cells with proper morphology. If possible, we selected well-fenestrated LSEC using cell membrane staining and without activated stress fibres (actin staining). For some inhibitors, as ML-7 or CalA, where cells are defenestrated, we search for representative and spread cells.
b) We use the same imaging parameters for all samples to compare the signal of pMLC or ppMLC calculated for 15-20 individual LSEC.

The method allowed for semiquantitative comparison between the groups of LSEC treated with different inhibitors. We still observed variations between animals, but all three experiments gave the

same trends. Moreover, super resolution approach allowed for detailed visualization of antibody distribution in single cell. For example, similar, elevated levels of ppMLC were observed in CytB and CalA treatments. However, the distribution of antibody was highly different and together with imaging of actin fibers indicated the distinct effect on fenestrations (F**igures S2 and S3**).

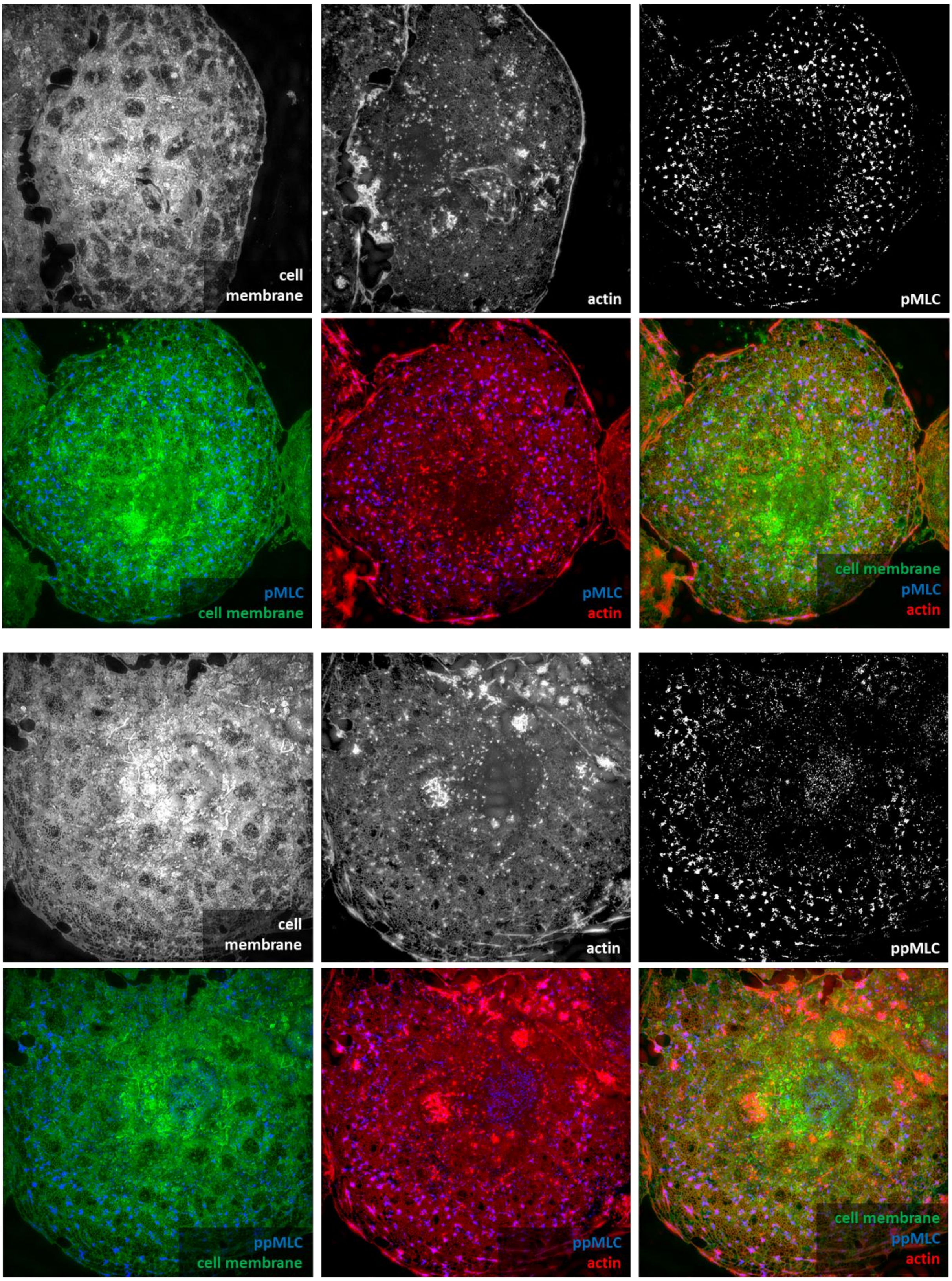

Figure S2| *CytB effect on pMLC/ppMLC in LSEC. Cell membrane (CellMask orange) and actin (phalloidin-Atto647N) were used to colocalize antibody with cell features e.g., actin dots. Image size: 40.96 µm x 40.96 µm.*

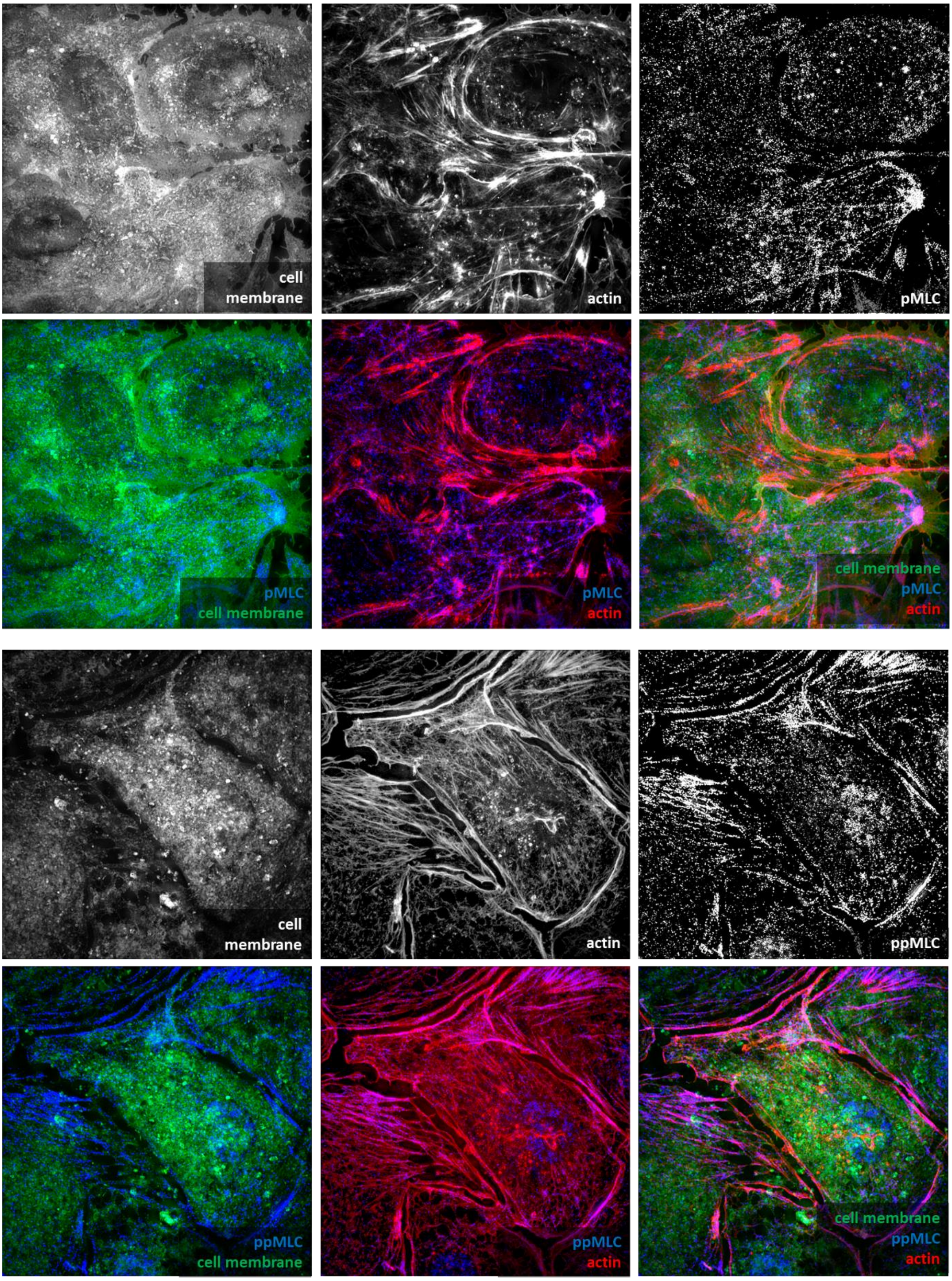

Figure S3 | *CalA (100 nM) effect on pMLC/ppMLC in LSEC. Cell membrane (CellMask orange) and actin (phalloidin-Atto647N) were used to colocalize antibody with cell features, e.g., stress fibres. Image size: 40.96 µm x 40.96 µm.*

SIM data provided information about cumulative effect of selected inhibitors. For example Pre-treatment with CytB or blebbistatin partially rescued LSEC from the contractile effect of CalA (100nM), allowing some fenestrations to remain opened (**Figure S4**).

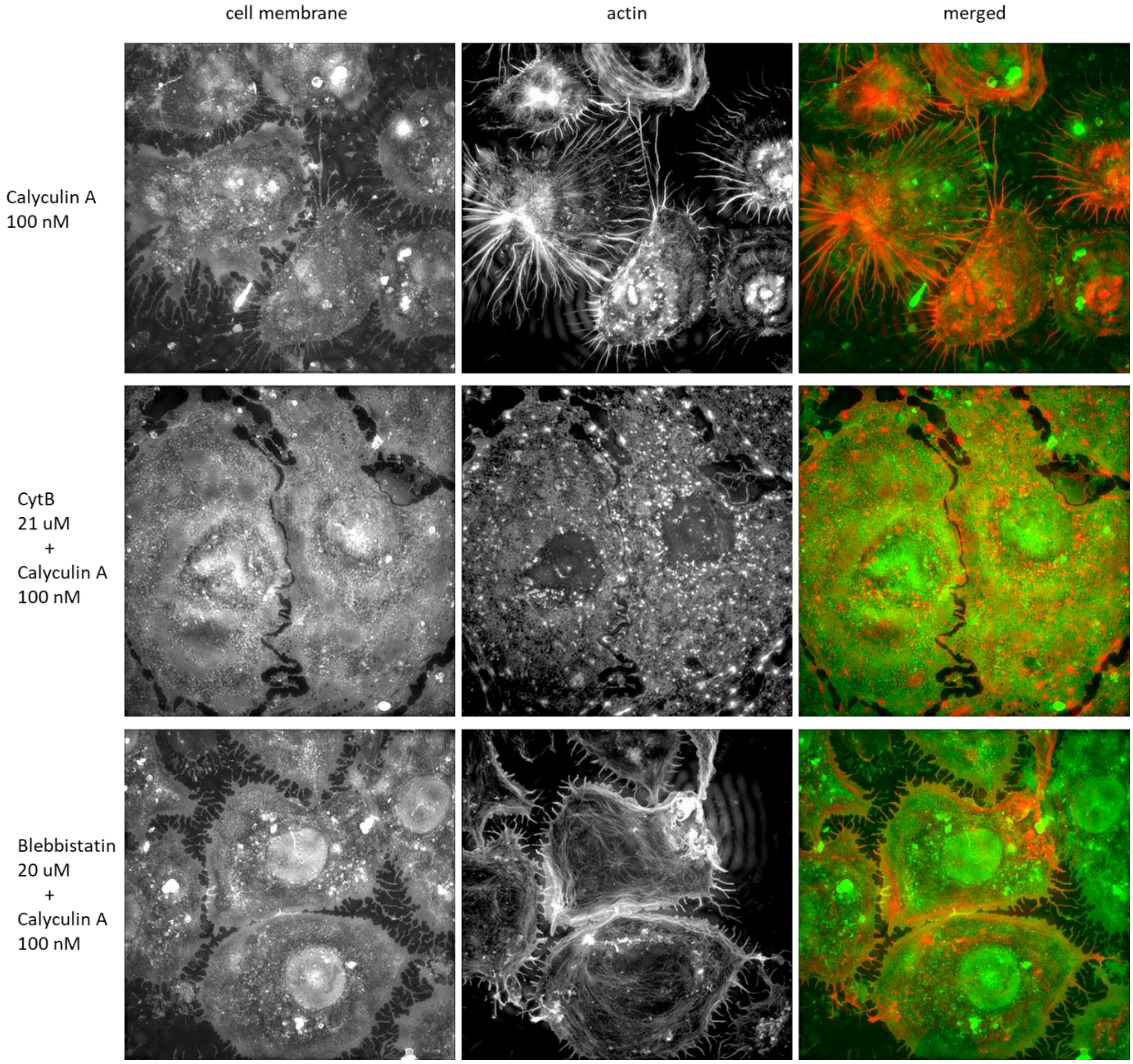

Figure S4 *| CalA (top row), CytB+CalA (middle row), and blebbistatin+CalA (bottom row) effect on LSEC. Cell membrane (CellMask green) and actin (phalloidin-Atto647N). Image size: 40.96 µm x 40.96 µm*

Finally, SIM was used to verify the effect of a cumulative effect of blebbistatin and CytB (**Figure S5**). LSEC were treated for 1h with 20 µM blebbistatin (the effect of blebbistatin was presented in Figure 4 and 6) followed by 30 min CytB (21 µM) before fixating and labelling. We observed an effect similar to CytB alone.

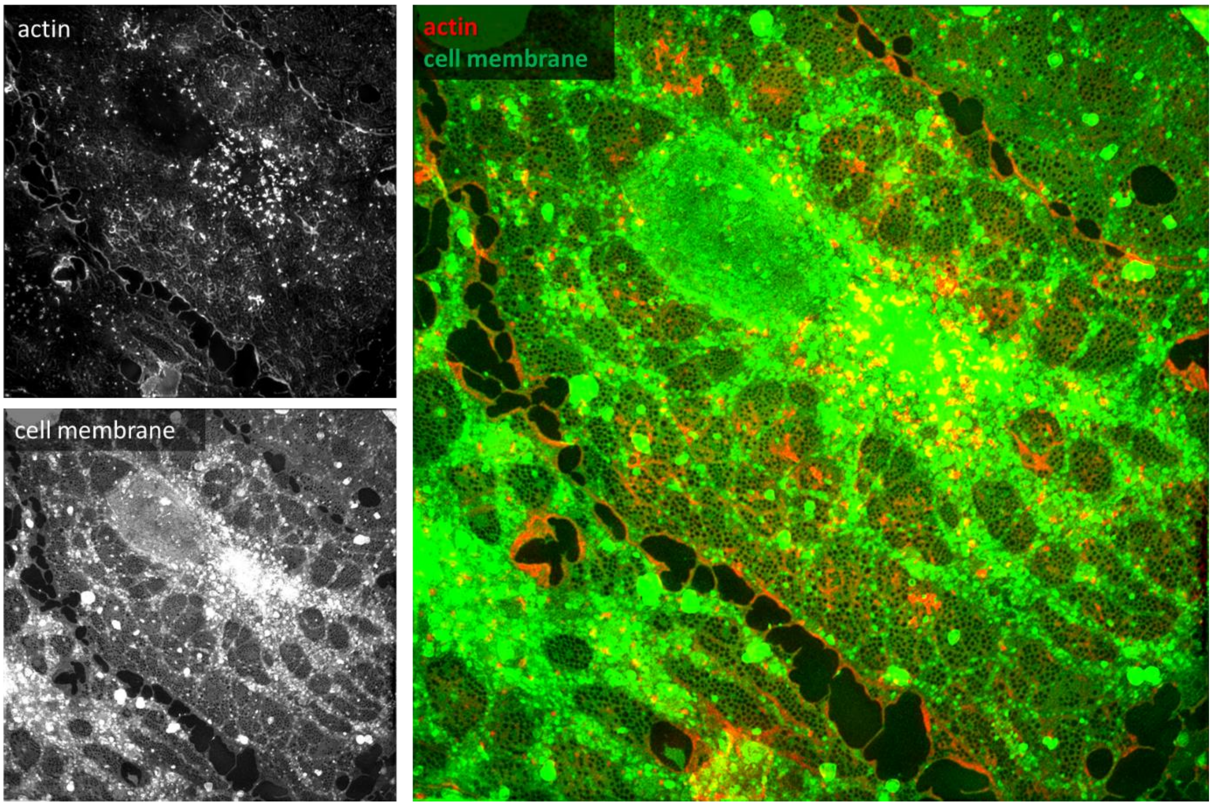

***Figure S5** | The cumulative effect of blebbistatin (20 µM, 60 min) and CytB (21 µM, 30 min). samples were fixed after the treatment and labelled with phalloidin-Atto 647N and Cell mask green. Image size: 40.96 µm x 40.96 µm.*

3. Semi-quantitative LSEC morphology analysis using overview mode in SEM

In general, LSEC porosity is calculated from single cell analysis. Usually, several representative cells are ramdomly selected and analysed in detail. Such an approach has been applied in research of LSEC morphology over the years. It is mainly because of the limitation of a maximum field of view in the microscopy techniques. If representative cells are selected for each treatment group, then the observed changes can be compared quantitatively. However, when the changes are small, the results can be biased by the selection of cells. Application of high end SEM microscope enable acquisition of overview images (about 300x magnification), where 50-100 cells can be visualized in a single image with a resolution enough for assessment of LSEC morphology (**Figure S6**).

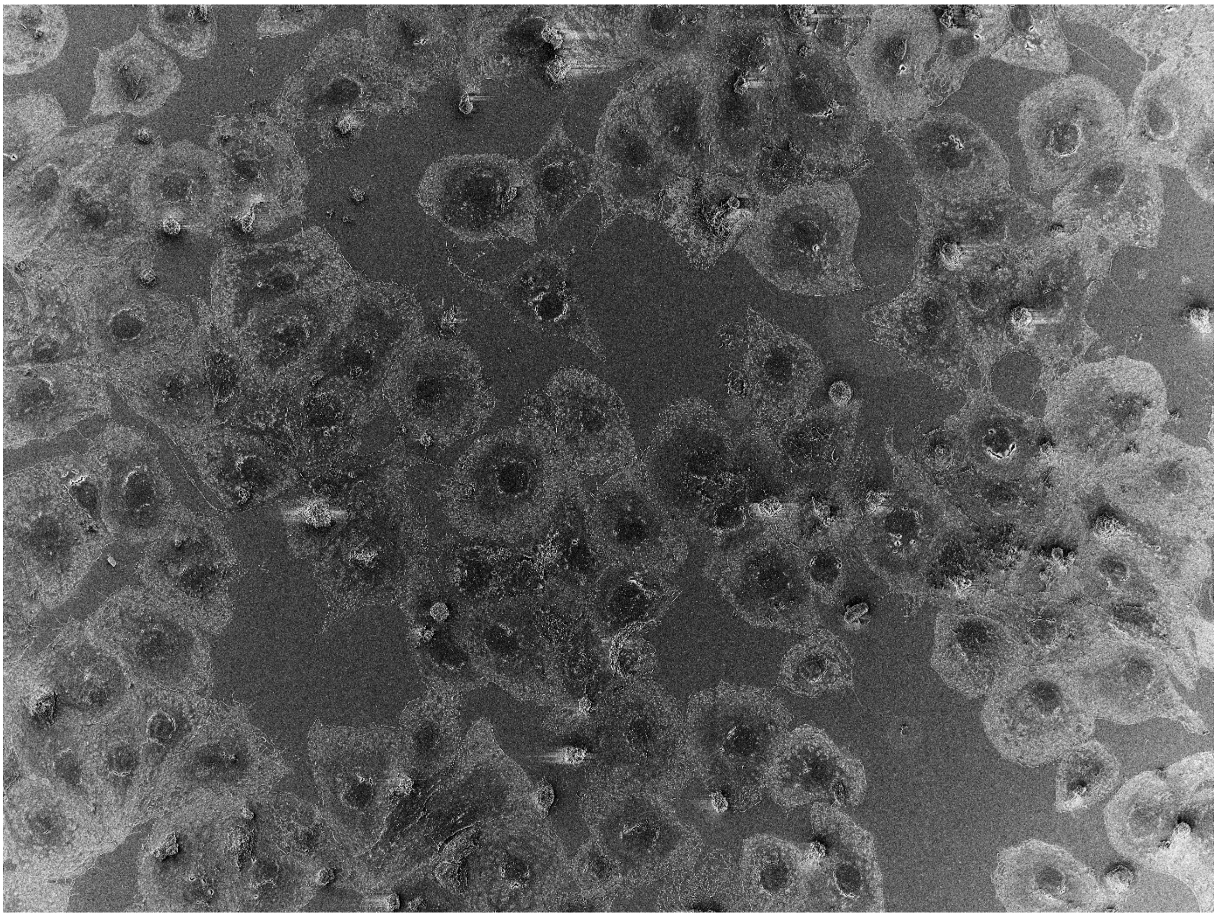

Figure S6 | An overview of control (untreated) LSEC sample. Magnification of 300x.

The resolution does not allow for proper quantification of fenestrations; however, it provides an additional information of the whole population of LSEC. LSEC were subjectively assigned with one out of five groups:

1. Highly fenestrated cells – nearly the whole cell body is covered in fenestrations, enlarged sieve plates, borders between sieve plates difficult to detect.
2. Normally fenestrated cells –regularly fenestrated cells, regular, easily distinguishable sieve plates.
3. Low fenestrated cells – cells with just few sieve plates/fenestrations that can barely be distinguished as LSEC.
4. Defenestrated cells – cells without fenestrations but with intact cell body.
5. Shrunken cells – cells with retracted cytoplasm, but without any morphological signs of apoptosis or necrosis, characteristic after treatment with calyculin A.

Figure S7 shows examples of cells assigned with each category.

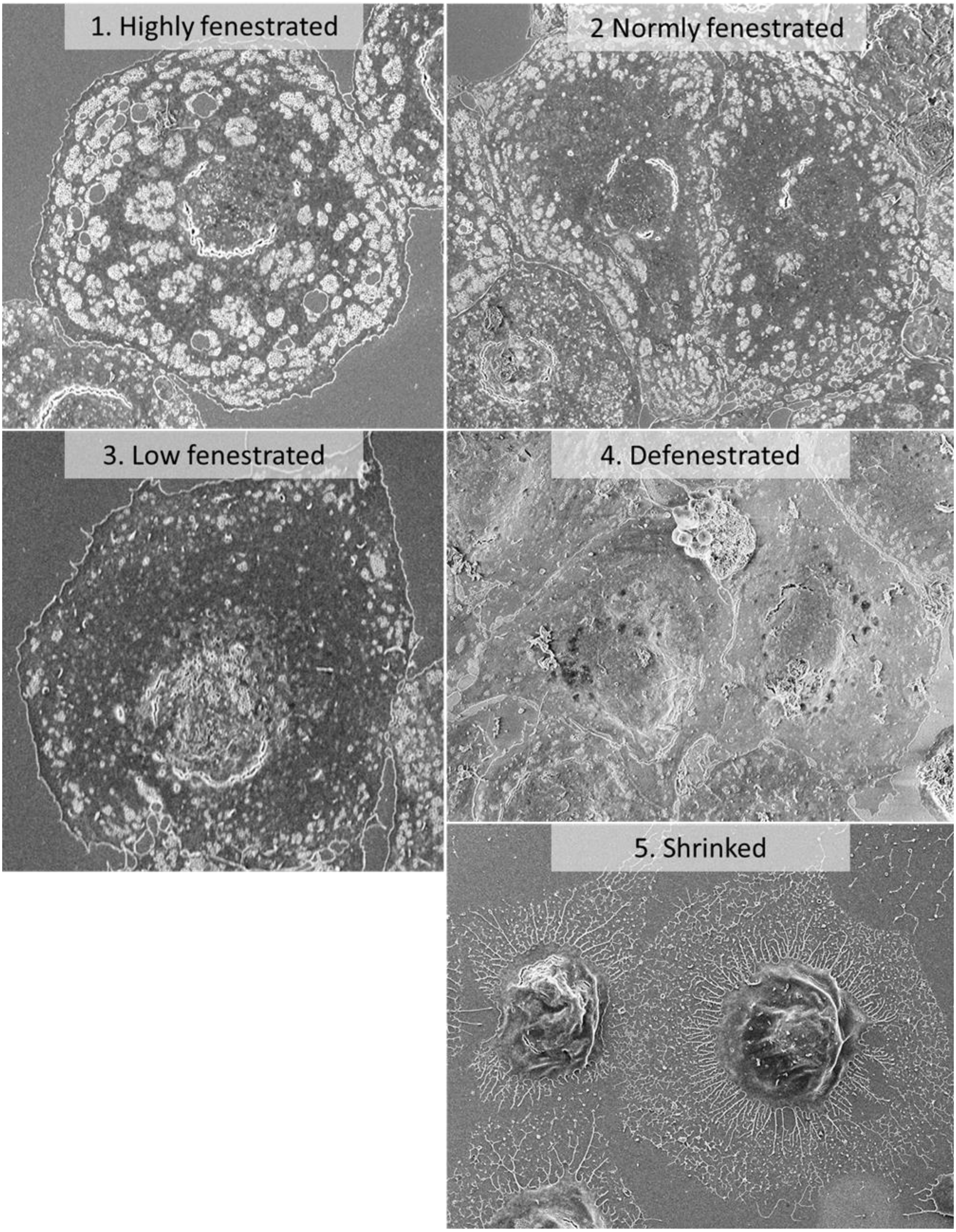

Figure S7| *Example images of LSEC assigned with one from five manually assigned categories. Each image is a zoom from the overview image, such as Figure S6.*

The whole analysis was performed by an experienced single user in a single scoring session to avoid bias. Each cell was individualy assigned and the final results (**Figure S8**) combine the data from three overview images from each of three experiments. Cells with clearly visible sample preparation artifacts (such as removed nuclei) or signes of necrosis/apoptosis were excluded from the analysis.

This method allow to quickly semi-quantitatively describe the whole population of cells and is compementary to the detailed fully quantitative analysis used to calculate LSEC morphology parameters – fenestration diameter/ferquency and porosity.

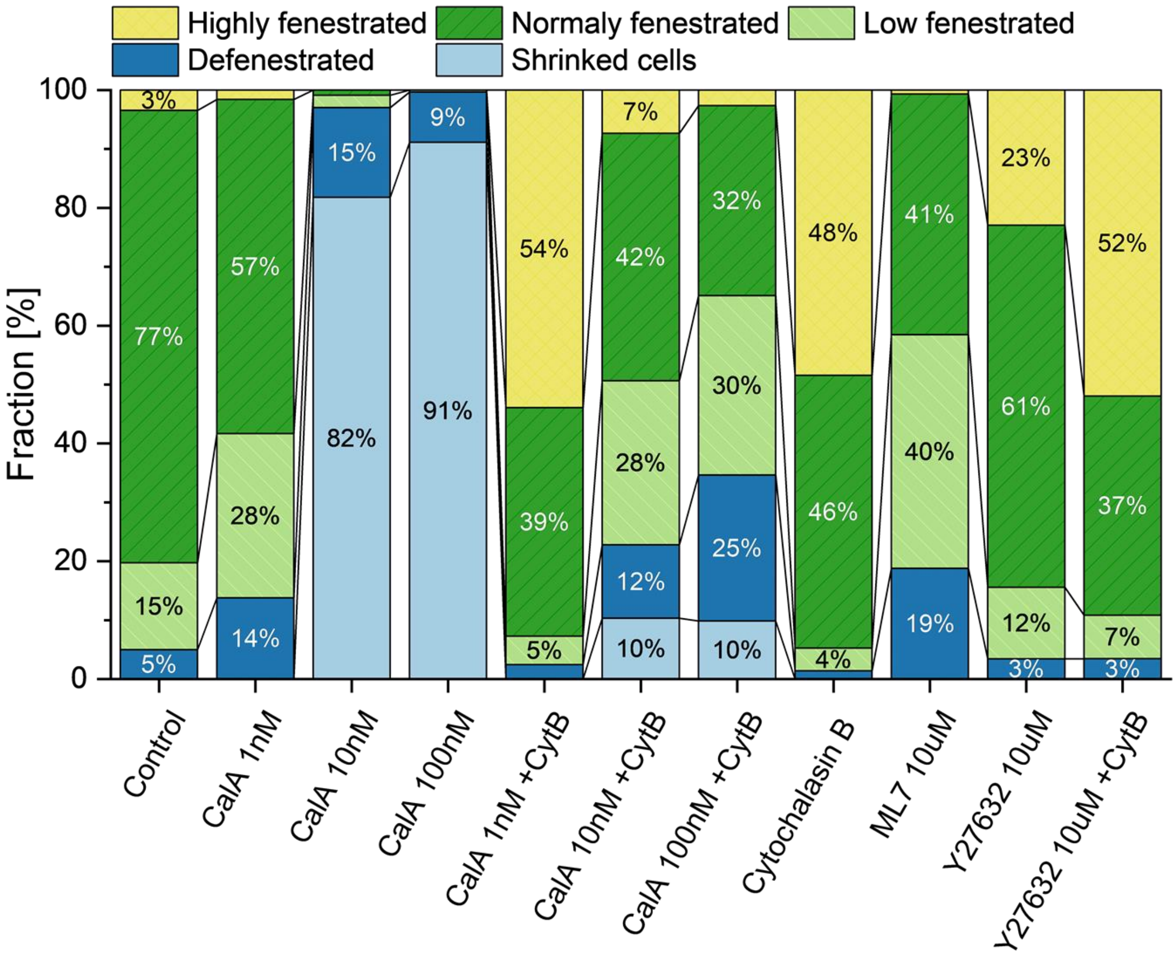

Figure S8 *| Fraction analysis of LSEC treated with selected inhibitors. Fractions <3% were not indicated*

4. Relaxed versus stretched fenestrations

We observed that reduction of actin filaments and pMLC relaxation via ROCK resulted in smaller fenestrations diameters. The opposite effect of thicker actin filaments, high level of MLC phosphorylation was in pair with larger fenestration, e.g. After CalA+CytB. As MLC antibody was observed on the edges of sieve plates and not within them, we believe that the regulation of fenestration size by MLC may occur at the level of a whole sieve plate, with a passive actin net within. We suggest that the contraction of actomyosin at the edges of sieve plate causes its contraction (increased tension) and enlargement, resulting in dilated fenestrations (actin net) within. The opposite effect of MLC dephosphorylation causes actomyosin relaxation. In combination with formation of thin actin mesh it results in large number of small fenestrations (**Figure S9**).

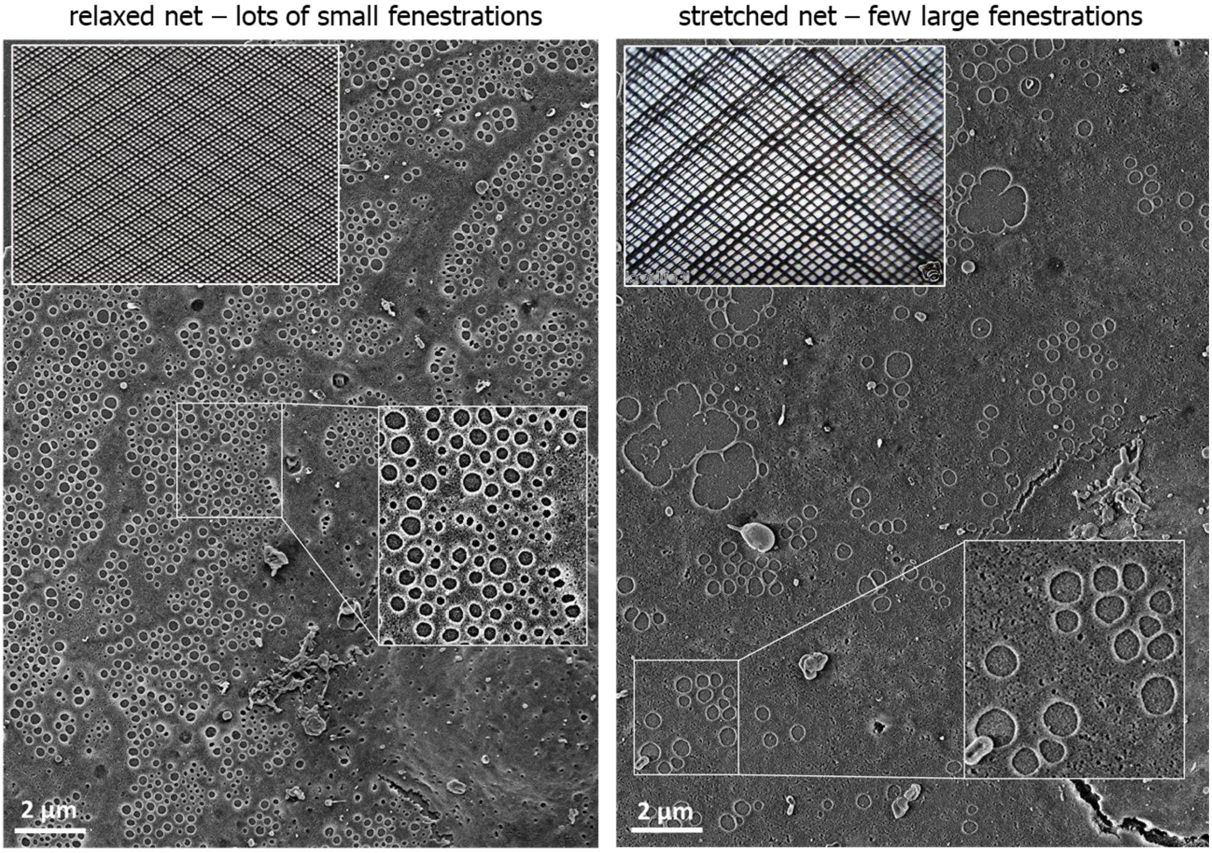

***Figure S9**| Schematic representation of a relaxed and stretched net corresponding to large number of small fenestrations and small number of large fenestrations after Y27632+CytB and CalA+CytB treatment, respectively.*

5. The effect of Calyculin A on the morphology of LSEC

In addition to Figure 2 of the main manuscript, here we provide representative images of LSEC treated with different concentration of CalA with and without pre-treatment with CytB (**Figure S10**). Here, we could not reproduce the experiment in the same way as for Y27632, i.e., pre-treatment with the agent and then treatment with CytB. CalA in concentration higher than 1 nM caused cell shrinkage and fenestrations could be observed only in a few cells that remained flat. This observation is illustrated in Figure S8. Here we present the reversed situation, where LSEC were treated first CytB then CalA. It prohibited the contractile effect of CalA, but no new fenestrations were formed due to CalA. Therefore, the contractile effect of CalA was attenuated. In our hands pre-treatment with CytB prevents the contractile effect of CalA for 1 nM and 10 nM and only 100 nM concentration of CalA had a significant effect on fenestration diameters. Nevertheless, we observed a gradually increasing contribution of fenestrations >200 nm for all concentrations.

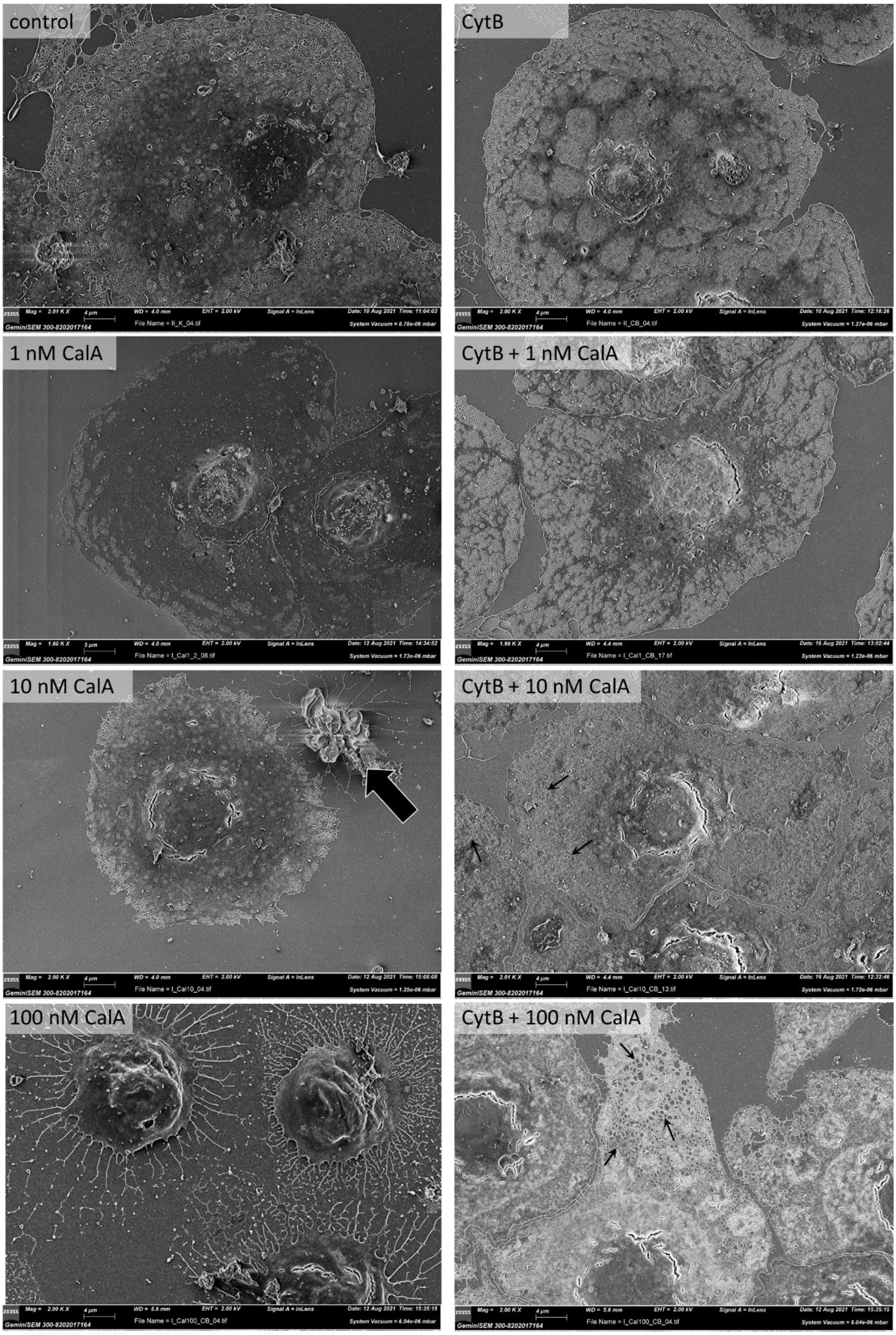

Figure S10 | *Representative SEM images of LSEC treated with different concentration of CalA with or without pre-treatment with CytB. 10 nM CalA causes majority of cells to shrink (thick arrow); 100 nM CalA resulted in shrinkage of all LSEC. Pre-treated (with CytB) LSEC, treated with 10 nM and 100 nM CalA present the presence of large fenestrations (arrows) and reduced number of fenestrations.*

6. The list of reagents used in the experiments.

Supplementary Table S1 | The list of reagents used in the experiments

REAGENT ; (abbreviation used in the article)	SOURCE	CATALOGUE NUMBER
INHIBITORS/DRUGS		
Y-27632 dihydrochloride ; (Y27632)	Merck, Darmstadt, Germany	Y0503
calyculin A ; (CalA)	BioNordika AS / Cell Signaling Technology, Oslo, Norway	#9902s
(-)-Blebbistatin ; (blebbistatin)	Merck, Darmstadt, Germany	B0560
ML 7 hydrochloride ; (ML7)	Bio-techne, Abbington, United Kingdom	#4310
KN 93 ; (KN93)	Merck, Darmstadt, Germany	K1385
cytochalasin B ; (CytB)	Merck, Darmstadt, Germany	C2743
DYES/ANTIBODIES		
CellMask green ; (CMG)	Thermo Fisher Scientific, Oslo, Norway	C37608
CellMask orange (CMO)	Thermo Fisher Scientific, Oslo, Norway	C10045
phalloidin-Atto647	Merck, Darmstadt, Germany	65906
Phospho-Myosin Light Chain 2 (Thr18/Ser19) Antibody	BioNordika AS / Cell Signaling Technology, Oslo, Norway	#3674
Goat Anti-Rabbit IgG H&L (Alexa Fluor® 488)	Abcam, Cambridge, United Kingdom	ab150077
Phospho-Myosin Light Chain 2 (Ser19) Antibody	BioNordika AS / Cell Signaling Technology, Oslo, Norway	#3671
OTHER		
ProLong™ Gold	Thermo Fisher Scientific, Oslo, Norway	P10144
Resazurin assay	R&D Systems, Abingdon, United Kingdom	AR002
LDH-Glo™ cytotoxicity assay	Promega Biotech AB, Nacka, Sweden	J2380

Suppl. Bibliography:

1. Blomhoff, R., Eskild, W. & Berg, T. Endocytosis of formaldehyde-treated serum albumin via scavenger pathway in liver endothelial cells. *Biochem. J.* **218**, 81–86 (1984).
2. Sørensen, K. K. *et al.* The scavenger endothelial cell: A new player in homeostasis and immunity. *Am. J. Physiol. - Regul. Integr. Comp. Physiol.* **303**, (2012).
3. Bhandari, S., Larsen, A. K., McCourt, P., Smedsrød, B. & Sørensen, K. K. The Scavenger Function of Liver Sinusoidal Endothelial Cells in Health and Disease. *Front. Physiol.* **12**, 1–23 (2021).
4. Simon-Santamaria, J. *et al.* Age-related changes in scavenger receptor-mediated endocytosis in rat liver sinusoidal endothelial cells. *J. Gerontol. A. Biol. Sci. Med. Sci.* **65**, 951–60 (2010).
5. Mccuskey, R. S. Sinusoidal endothelial cells as an early target for hepatic toxicants. **34**, 5–10 (2006).

6. Politz, O. *et al.* Stabilin-1 and -2 constitute a novel family of fasciclin-like hyaluronan receptor homologues. *Biochem. J.* **362**, 155–164 (2002).
7. Hansen, B. *et al.* Stabilin-1 and stabilin-2 are both directed into the early endocytic pathway in hepatic sinusoidal endothelium via interactions with clathrin/AP-2, independent of ligand binding. *Exp. Cell Res.* **303**, 160–173 (2005).
8. Mccourt, P. A. G. Characterization of a Hyaluronan Receptor on Rat Sinusoidal Liver Endothelial Cells and Its Functional Relationship to Scavenger Receptors. *Hepatology* 1276–1286 (1999).
9. Kus, E. *et al.* LSEC fenestrae are preserved despite pro-inflammatory phenotype of liver sinusoidal endothelial cells in mice on high fat diet. *Front. Physiol.* **10**, 1–14 (2019).
10. Martinez, I. *et al.* The influence of oxygen tension on the structure and function of isolated liver sinusoidal endothelial cells. *Comp. Hepatol.* **7**, 1–11 (2008).
11. Sebbagh, M. *et al.* Caspase-3-mediated cleavage of ROCK I induces MLC phosphorylation and apoptotic membrane blebbing. *Nat. Cell Biol.* **3**, 346–352 (2001).
12. Fazal, F. *et al.* Inhibiting Myosin Light Chain Kinase Induces Apoptosis In Vitro and In Vivo. *Mol. Cell. Biol.* **25**, 6259–6266 (2005).
13. Zapotoczny, B. *et al.* Tracking Fenestrae Dynamics in Live Murine Liver Sinusoidal Endothelial Cells. *Hepatology* **69**, 876–888 (2019).
